# Stress and exhaustion among medical students: a prospective longitudinal study on the impact of the assessment period on medical education

**DOI:** 10.1186/s12909-024-05617-6

**Published:** 2024-06-06

**Authors:** Isabela Silva Santiago, Safira de Castro e Castro, Ana Paula Amaral de Brito, Daniel Sanches, Luiz Fernando Quintanilha, Katia de Miranda Avena, Bruno Bezerril Andrade

**Affiliations:** 1 Zarns Medical College, Clariens Education, 3230 Luís Viana Filho Avenue, Salvador, Bahia 41720-200 Brazil; 2 University of the State of Bahia, 2555 Silveira Martins Street, Salvador, Bahia 41150-000 Brazil; 3 Natural Sciences Department, Arts and Sciences Division, South Florida State College, 600 West College Drive, Avon Park, FL USA; 4 Laboratory of Clinical and Translational Research, Gonçalo Moniz Institute, Oswaldo Cruz Foundation, 121 Waldemar Falcão Street, Salvador, Bahia 40296-710 Brazil

**Keywords:** Medical students, Psychological stress, Medical education

## Abstract

**Background:**

Stress significantly affects both the physical and emotional health of individuals, particularly students in health-related fields. Medical students in Brazil face unique challenges due to the demanding nature of their studies, especially during assessment periods, which heighten academic pressure. These pressures often lead to poor coping strategies and mental health concerns. It is crucial to understand the complex dynamics of stress within medical education to develop strategies that improve student well-being and promote a healthier academic environment. This study aims to investigate the intricate relationship between assessment periods and stress levels among medical students. It seeks to understand how academic demands and sociodemographic factors contribute to stress dynamics during these periods.

**Methods:**

An online observational, longitudinal, and prospective study was conducted from February to October 2022. Medical students were recruited through snowball sampling and participated in surveys administered via Google Forms at two timepoints: before (T1) and during (T2) assessment periods. The surveys collected sociodemographic data and stress symptoms using Lipp’s Inventory of Stress Symptoms for Adults (LSSI).

**Results:**

The transition from T1 to T2 was defined by a rise in the prevalence of stress from 59.6 to 84.2% (*p* = 0.001) and a decline in symptom-free students from 40.4 to 15.8% (*p* = 0.001). There was a significant increase in exhaustion, from 12.3 to 31.6% (*p* = 0.0001). Higher stress levels were notably more prevalent among younger students (≤ 24 years), females, those from wealthier families, students without scholarships, those without prior degrees, and those in the clinical phase of their studies. However, non significant correlations were found between these sociodemographic and academic factors and the increase in stress.

**Conclusion:**

The findings highlight significant concerns regarding the mental health of medical students during assessment periods, marked by increased stress and exhaustion levels. These results emphasize the need for proactive interventions to manage stress effectively in medical education, considering its profound impact on students’ well-being.

## Background

Stress, as the body’s response to various stimuli triggering psychophysiological adaptation, profoundly influences an individual’s physical and emotional well-being. These stimuli can wield either positive or negative impacts, yet their persistence over time can significantly deteriorate an individual’s mental and physical health [1].

In recent years, several studies have illuminated a troubling trend: health profession students exhibit markedly higher rates of mental illness compared to the general population [2–5]. This concerning observation highlights the unique challenges faced by those pursuing careers in health-related fields.

Among these professions, medicine stands out for its notorious blend of heavy workload and high degree of self-demand, which requires an intense routine and arduous dedication to academic pursuit [6].

Within the challenging environment of medical school, students contend with a myriad of stressors capable of disrupting the delicate balance between their academic and personal lives [7]. These stressors frequently manifest in nonadaptive coping mechanisms [7], potentially stemming from challenges in acknowledging and confronting their fears [7–9]. This exacerbates the susceptibility to debilitating mental health conditions, including anxiety, depression, general mental disorders, and burnout [10, 11].

In Brazil, some educational institutions adopt a specific interval within the academic calendar during which students undergo evaluations, examinations, or assessments of their academic performance. Within this context, the assessment period stands out as a temporal crucible within the university environment. This period, characterized by an intensified need for academic commitment, brings with it a cascade of challenges that reverberate through students’ lives [12]. It is a time marked by relentless self-critique, the burden of study overload, sleep deprivation, and a pervasive sense of isolation from familial and social support networks [13].

Faced with increasing academic pressures, students may resort to inappropriate coping strategies to increase their study time and performance [14], further compounding their psychological distress. The pursuit of academic excellence often collides with the erosion of well-being, raising questions about the true pedagogical value of assessments in such an environment.

In light of these pressing concerns, this study aimed to explore the nuanced interplay between the assessment period and stress levels among medical students. By examining the associations with academic and sociodemographic profiles, we aim to elucidate the multifaceted landscape of stress within the medical education environment.

## Methods

### Study design and sampling methods

To achieve the study’s objectives, an online observational, longitudinal, and prospective study was conducted between February to October 2022.

At the beginning of the 2022 academic year, all medical students from a private medical shool in Brazil (*n* = 906) were invited to participate in the study through virtual messages sent via instant messaging applications. The snowball sampling methodology was adopted for the recruitment of participants. This non-probabilistic technique is frequently used in virtual research to recruit participants with specific characteristics. The process allows participants themselves to recruit other participants who are also suitable for the study, allowing them to gradually expand the sample size [15, 16]. The response rates were 19,4% (*n* = 176).

### Participants

The study involved undergraduate medical students at Shool of Medicine from Salvador, Bahia, Brazil. Participants were recruited if they met the following inclusion criteria: (a) aged 18 years and above; and (b) regularly enrolled in the course. Students who did not respond to the questionnaire at both scheduled times were excluded.

### Measurements

Data collection occurred in 2022, being carried out at two timepoints: before (T1) and during (T2) the assessment period of the first unit of each academic semester (2022.1 and 2022.2). For the 2022.1 semester, T1 occurred in February, while T2 occurred in April, while for the 2022.2 semester, T1 and T2 occurred in the months of August and October, respectively. We collected the data by administering student questionnaires via Google Forms.

During T1, participants completed a sociodemographic questionnaire covering age, sex, academic cycle, scholarship or student financing status, and prior graduation. Additionally, the Lipp’s Inventory of Stress Symptoms for Adults (LSSI) [17] was administered at both T1 and T2. This inventory comprises three parts with objective questions identifying physical and psychological stress symptoms, enabling differentiation into four phases: alertness, resistance, near exhaustion, and exhaustion.

The first part of LSSI comprised 12 physical and three psychological symptoms experienced in the past 24 h; the second part included 10 physical and five psychological symptoms from the previous week, while the third part encompassed 12 physical and 11 psychological symptoms from the last month. Notably, some symptoms from the first part reappeared with increased intensity in the third part [17]. LSSI consists of 53 items, 34 of which are somatic and 19 psychological. A positive stress diagnosis is determined based on the cumulative symptoms reported in each inventory part [17].

### Data analysis

The data were analyzed using IBM SPSS statistical software, version 25.0. In terms of descriptive statistics, categorical variables were presented as the distribution of categorical frequencies, expressed in absolute numbers and percentages. The numerical variables were presented as arithmetic means and standard deviations when they had a normal distribution and as medians and interquartile ranges when they had an non-Gaussian distribution.

The analyses were conducted on both the entire sample and on groups of students categorized based on their current academic cycle. The academic cycle was divided into two main groups: the basic cycle, comprising 1st and 2nd years, and the clinical cycle, encompassing 3rd and 4th years. This subgrouping analysis is important because students experience distinct challenges within each academic phase, potentially resulting in different influences on their stress levels.

For analytical statistics, we applied the Kolmogorov‒Smirnov normality test for quantitative variables. The unpaired t test and the Mann‒Whitney *U* test were used to compare continuous variables between groups, and the chi‒squared test was used for categorical variables. Compared with the sociodemographic and academic variables, the stress variable was dichotomized according to whether it had increased. Comparisons between the “before” (T1) and “during” (T2) assessments were performed using the nonparametric Wilcoxon signed rank test for paired samples.

To assess changes in stress levels and the presence of symptoms between the two study timepoints, we created two variables called “stress behavior” and “symptoms behavior”, representing the difference between T2 and T1 assessments. Results were categorized as increased, maintained, or decreased of stress or symptoms. Increased stress was considered the primary outcome. To analyze Tab1associations between sociodemographic and academic variables and increased stress, we used odds ratios (ORs), contingency coefficients, and chi-square tests with Bonferroni post hoc correction for multiple comparisons. For all analyses, the level of statistical significance was set at 0.05 or 5%.

### Ethics approval and consent to participate

The study complied with Resolution 466/12 of the Brazilian Health Council and was approved by the Brazilian Research Ethics Committee (protocol No. 44150621.7.0000.5032).

The participants’ autonomy, confidentiality, and privacy were carefully maintained throughout the study. Data confidentiality and participant privacy were ensured by restricting information access to the involved researchers exclusively. Each participant was assigned a unique random number, and responses were matched using the first three letters of their first name and the initial three digits of their Brazilian Individual Taxpayer Identification Number. To uphold the principle of autonomy, participants were provided access to the data collection instrument via a link distributed by the researchers before completing the survey. This allowed them to review the questionnaire items before deciding whether to participate in the study. These stringent measures were implemented by ethical principles and research guidelines, aiming to safeguard participant well-being and ensure the scientific validity of the study. These measures are also in compliance with the Brazilian General Personal Data Protection Law. Moreover, all study participants were fully informed about the research objectives and methods and provided their consent by signing the informed consent form.

## Results

A total of 176 students responded to the survey. One hundred forty-five medical students participated in the study during the initial data collection (T1), and 88 participated in the subsequent stage (T2). Among this cohort, only 57 students completed the questionnaire at both time points, thus composing the final study population.

Most of the study participants were female (77.2%), with a mean age of 24.0 ± 4.7 years and a monthly family income ranging from 3 to 9 times the minimum wage (29.8%). Regarding academic aspects, the majority were in the clinical cycle (42.1%), did not hold a previous degree (80.7%), and did not receive student grants or funding (59.6%) (Table 1).

**Table 1:** Baseline characteristics of the study population

Characteristics	All (*n* = 57)	Academic cycle		*p*-value**
		Basic (*n* = 24)	Clinical (*n* = 33)	
Age, AM *±* SD (years)	24.0 *±* 4.7	22.8 *±* 4.8	24.9 *±* 4.5	0.763^§^
Sex, n (%)				0.736^¥^
Female	44 (77.2)	18 (75.0)	26 (78.8)	
Male	13 (22.8)	6 (25.0)	7 (21.2)	
Family income, n (%)				0.378^¥^
I do not know	9 (15.8)	4 (16.7)	5 (15.2)	
Up to 3 minimum wages	11 (19.3)	3 (12.5)	8 (24.2)	
3 to 9 minimum wages	17 (29.8)	7 (2962)	10 (30.3)	
9 to 12 minimum wages	5 (8.8)	2 (8.3)	3 (9.1)	
Above 12 minimum wages	15 (26.3)	8 (33.3)	7 (21.2)	
Student Scholarship*, n (%)	23 (40.4)	10 (41.7)	13 (39.4)	0.863^¥^
Previous degree, n (%)	11 (19.3)	5 (20.8)	6 (18.2)	0.802^¥^
Total symptoms, MD (IIq 25–75)				
Before Evaluation	11 (7–18)	12 (8–14)	14 (10–17)	0.246^£^
During Evaluation	19 (11–26)	19 (15–22)	19 (15–23)	0.348^£^
Increase in Symptoms, n (%)	43 (75.4)	22 (51.2)	21 (48.8)	0.057^¥^
Prevalence of Stress, n (%)				
Before Assessment - T1	34 (59.6)	13 (54.2)	21 (63.6)	0.472^¥^
During Assessment - T2	48 (84.2)	20 (83.3)	28 (84.8)	0.877^¥^
Increase in stress, n (%)	26 (45.6)	12 (50.0)	14 (42.4)	0.571^¥^

*n* absolute number; %: percentage; AM: arithmetic mean; SD: standard deviation; MD: median; IIq 25–75: Interval calculated from the 25th and 75th percentiles of the data; *Financial aid granted to students by Brazilian government programs such as the University for All Program (Prouni) and the Higher Education Student Financing Fund (FIES); **Comparison between academic cycles; §Unpaired t test; ¥Chi-square test; £Mann‒Whitney test.]

Despite the distinct challenges experienced in each academic cycle, no statistically significant differences were found between the groups of students in the basic or clinical cyle, with regard to the evaluated features emcompassing sociodemographic, academic, or stress characteristics (Table 1). This suggests that the subgroups of students based on academic cycle appeared to have similar characteristics regardless of when they completed the questionnaires. Consequently, the further analyzes concatenated all the medical students primarily included in the research, without the necessity of dividing them into subgroups based on the ongoing academic cycle.

When comparing the data from T1 and T2, there was an increase in the prevalence of stress (T1: 59.6% vs. T2: 84.2%, *p* = 0.001) and a decrease in the number of students reporting no physical or psychological symptoms of stress (T1: 40.4% vs. T2: 15.8%, *p* = 0.001). Additionally, a significant rise was observed in the proportion of students experiencing exhaustion (T1: 12.3% vs. T2: 31.6%, *p* = 0.0001) (Table 2).

**Table 2:** Analysis of medical students’ stress levels and associations with the assessment period (n=57)

Stress	Assessment period		p*
	T1	T2	
Stress			**0.001**
Present	34 (59.6)	48 (84.2)	
Absent	23 (40.4)	9 (15.8)	
Level, n (%)			**0.0001**
No stress	23 (40.4)	9 (15.8)	
Resistance	25 (43.9)	26 (45.6)	
Near exhaustion	2 (3.5)	4 (7.0)	
Exhaustion	7 (12.3)	18 (31.6)	

*n* absolute number; %: percentage; *Wilcoxon test with Bonferroni post hoc correction

During the evaluation period, heightened stress levels were predominantly observed among students aged up to 24 (73.1%), females (76.9%), with a family income exceeding 12 times the minimum wage (26.9%), without a student grant (65.4%), lacking a previous degree (84.6%), and enrolled in the clinical cycle (53.8%). However, a logistic regression analysis including those variables did not show a significant association with increased stress in the study population (Table 3).

**Table 3:** Analysis of factors associated with increased stress during the assessment period among the medical students assessed

Crossings		OR (CI 95%)	Coefficient of contingency	*p* value§
Increased stress	Age > 24 years-old	0.45 (0.15–1.37)	0.109	0.409
	Female	1.02 (0.30–3.56)	0.376	0.052
	Basic Academic Cycle in medical school	0.74 (0.26–2.12)	0.095	0.472
	Student Scholarship*	0.64 (0.22–1.88)	0.052	0.692
	Previous academic degree**	0.62 (0.16–2.42)	0.040	0.764
	Increase in symptoms	0.77 (0.22–2.69)	0.054	0.684

OR (95% CI): odds ratio with 95% confidence interval. *Financial aid granted to students by Brazilian government programs such as the University for All Program (Prouni) and the Higher Education Student Financing Fund (FIES); **Medical students that have other graduation degrees, which could be in any other subject; §Chi-square test with Bonferroni post hoc correction for multiple comparisons

## Discussion

Assessment plays a crucial role in undergraduate medical education, essential for students’ academic and professional development. It provides continuous feedback, identifies areas for knowledge improvement, and adapts teaching to individual and collective needs to promote deep, self-directed, meaningful, and motivated learning [18]. A well-designed assessment period evaluates theoretical knowledge, practical skills, ethics, and communication, essential for training competent and committed physicians for the challenges of clinical practice.

Several educational organizations advocate for a global reconsideration of the assessment period, promoting procedures that stimulate skill and competency development, as well as critical thinking [19–21]. Despite diverse assessment methods, multiple-choice tests continue to dominate medical education and selection processes [22], including Brazil’s essential examinations such as the National Undergraduate Student Achievement Examination and the National Residency Examination are based on this strategy. Therefore, medical students must be adequately prepared, both physically and psychologically, for these exams.

In this context, Sein, Dathatri, and Bates [18] proposed guidelines to assist stakeholders in medical education, emphasizing strategies to reduce exam-related anxiety and stress. Despite stress’s perceived neutrality on academic performance [23–26], its high prevalence among students warrants educators’ attention due to its potential physical and psychological impacts [17].

Our study revealed a notable stress prevalence among medical students (59.6%), with 15.8% experiencing “near exhaustion” or “exhaustion”. Consistent with national and international studies [12, 24, 27–30], stress factors include university adaptation, study methods, performance pressure, poor sleep quality, and financial concerns [25, 26, 28, 29, 31]. Although few studies explore the assessment period’s impact on stress, our findings suggest a negative influence, particularly among students on the verge of exhaustion.

While sociodemographic and academic characteristics did not correlate with increase in stress in our logistic regression analysis, it is important to highlight that younger and female students experienced higher stress levels. This trend is particularly significant in the context of medical education, characterized by feminization and juvenilization processes [32]. Medical education stakeholders should carefully consider this scenario, especially considering evidence of a higher prevalence of disorders among these demographic groups [24].

Despite the significant number of students experiencing stress, it’s essential to acknowledge that some remain unaffected, either before or during the assessment period. This underscores the significance of individual coping mechanisms and personal resilience in shaping one’s perception of stress. Strategies to reduce stress levels include valuing interpersonal relationships, time management, nutritional and sleep care, religious engagement, building support network, and seeking psychological assistance.

Neglecting student well-being poses individual and public health risks, impacting future physicians’ training and healthcare system users [10, 33]. Understanding stress causes and implications is crucial for proposing effective institutional strategies [34]. In addition, sensitizing students to their psychological aspects and reactions to course-related experiences is imperative. Medical schools must prioritize student care [7] and create safe and supportive environments permeated by respect, attentive listening, and acceptance to address diverse psychological demands throughout medical training.

### Strengths and limitations

The study’s limitations call for special attention. One potential limitation is the reduced participant adherence during the second phase of data collection, resulting in a significant exclusion rate of those who completed only the first phase of the study. Furthermore, it is important to consider the questionnaire’s length and the possibility of student fatigue during completion, factors that may have contributed to reduced participation, particularly during the exam week. Another relevant point is that the study was conducted at a single private medical school in Brazil, although it is the largest medical school in the country in terms of the number of medical students. This indicates that the findings described here require additional validation in other medical schools, ideally encompassing programs from various regions of the country, including public institutions. Regional disparities and the differing impacts of public versus private investment could influence stress levels differently, necessitating broader validation efforts to ensure the robustness and generalizability of the results.

Despite limited participant adherence during the second phase, our study provides longitudinal and prospective suggestions indicating the negative impact of the assessment period on stress and exhaustion among medical students. These findings prompt reflection on institutional intervention proposals aimed at alleviating the issue without compromising teaching quality and ensuring meaningful learning in future physicians’ training [35, 36].

Thus, the results underscore the urgent need for specific strategies and interventions targeting student groups identified as more vulnerable to increased stress. The medical education environment should implement preventive and supportive measures for students’ mental health, as although evidence regarding its impact on academic performance is insufficient, the significant increase in stress symptoms during the assessment period warrants reflection and action to mitigate the problem. These findings highlight the importance of personalized medical education approaches aiming not only for academic excellence but also for the holistic well-being of future health professionals.

## Conclusion

Together, the findings of the present study uncover worrying results regarding the mental health of medical students during the evaluation period, as medical students experienced a significant increase in the presence of stress and the incidence of exhaustion. Given the complexity and implications of stress in the educational context, especially in the demanding environment of medical courses, the results of this research highlight the relevance of a careful and proactive approach to understanding and mitigating the factors that amplify stress among students in this critical learning period.

## Data Availability

The datasets used and analysed during the current study are available from the corresponding author on reasonable request.

## Declarations

The study was approved by the Brazilian Research Ethics Committee (protocol no. 44150621.7.0000.5032) and all participants previously signed a written informed consent form (ICF).
